# Characterization of Corn Starch Films Reinforced with CaCO_3_ Nanoparticles

**DOI:** 10.1371/journal.pone.0106727

**Published:** 2014-09-04

**Authors:** Qingjie Sun, Tingting Xi, Ying Li, Liu Xiong

**Affiliations:** School of Food Science and Engineering, Qingdao Agricultural University, Qingdao, Shandong Province, China; Semmelweis University, Hungary

## Abstract

The characterization of corn starch (CS) films impregnated with CaCO_3_ nanoparticles was investigated. Criteria such as morphology, crystallinity, water vapor permeability (WVP), opacity, and mechanical properties were the focus of the investigation. It was found that the CaCO_3_ contents had significant effects on the tensile properties of the nanocomposite films. The addition of CaCO_3_ nanoparticles to the CS films significantly increased tensile strength from 1.40 to 2.24 MPa, elongation from 79.21 to 118.98%, and Young’s modulus from 1.82 to 2.41 MPa. The incorporation of CaCO_3_ nanoparticles increased the opacity of films, lowered the degree of WVP and film solubility value compared to those of the CS films. The results of scanning electron microscopy (SEM) showed that with the increase of CaCO_3_ nanoparticles content in starch films, the roughness of the films increased, and pores or cavities were found on the surface of the films, while small cracks were observed in the structures of the fractured surfaces. X-ray diffraction showed that the addition of nanoparticles increased the peaks in the intensity of films.

## Introduction

Starch has received considerable attention because of its totally biodegradable nature, low cost and wide availability [1]–[4]. Starch has been considered one of the biopolymers with the greatest potential to produce biodegradable films by different processing techniques such as casting, injection or blow molding and so on [5]. Several studies have reported the use of starches from different sources as raw material for films and coatings with different properties, showing the potential of this carbohydrate in these application fields [6]–[7]. However, there are some strong limitations to developing starch based films, which have poor tensile properties and high water vapor permeability on account of their hydrophilic nature and their sensitivity to moisture content. One possible approach to overcome this limitation is to strengthen starch matrixes with organic or mineral fillers [8]. These fillers reinforce biopolymeric matrixes and lead to the development of films with special properties due to the synergic effect between the components [9].

Nanoparticles are a new type of filler that show a higher level of efficiency in improving the physicochemical and mechanical properties of starch-based films. Nanoparticles have good compatibility with the matrix in the thermoplastic starch films [10]. The tensile and water barrier properties of cassava starch composite films are reinforced by synthetic zeolite and beidellite [11]. Besides, with the incorporation of the nanofiller, starch-based materials generally show improvement in thermal stability, oxygen barrier property, and biodegradation rate. [12]. Calcium carbonate (CaCO_3_) nanoparticles are a white powder and used as filler in composite materials, such as in plastics and in paper industry. However, there are no reports about starch films reinforced with CaCO_3_ nanoparticles.

The aims of this work are to develop nanocomposite films based on corn starch with CaCO_3_ nanoparticles and to evaluate the effect of filler addition on the mechanical properties and moisture resistance of corn starch/CaCO_3_ nanoparticles films.

## Materials and Methods

### Materials

**Figure 1:**
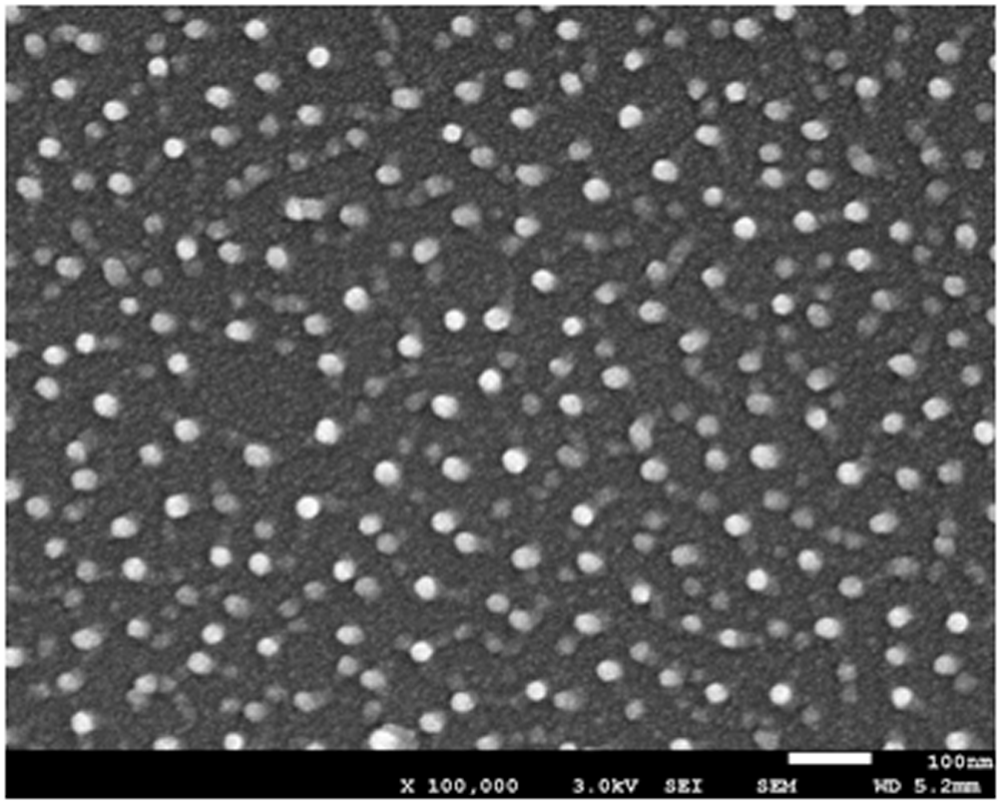
SEM micrographs (100000×) of CaCO_3_ nanoparticles.

Corn starch (amylose content 26.33%) was purchased from the National Starch Co. (Shanghai, China) and we obtained the CaCO_3_ nanoparticles, which have a high purity (98%), from Hefei Aiwei Nano Science and Technology Co., Ltd (Anhui, China). Fig. 1 shows the morphology and size of CaCO_3_ nanoparticles and the size is 35±2 nm. Glycerol (analytical grade) was used as plasticizer.

### Film preparation

The method of preparation was adapted from Araujo-Farro et al. (2010) with some modifications [13]. Corn starch (7.5 g) and glycerol (3.0 g) were added to 100 ml distilled water to obtain composite solutions. CaCO_3_ nanoparticles were dissolved in 50 ml distilled water by ultrasonic mixing. The percentage of the CaCO_3_ nanoparticles used was set at 0, 0.02, 0.04, 0.06, 0.1 and 0.5% levels on the dry basis of corn starch, respectively. It was mixed with the starch/glycerol composite solutions. The mixture solutions were heated in a boiling water bath with continuous agitation for 30 min to allow full gelatinization of the corn starch. After the heating period, the composite solutions were degassed under a vacuum (0.1 MPa) for 10 min and cooled down to room temperature. The mixture (about 65 g) was then spread evenly over Petri dishes (15 cm diameter) and evaporated in a ventilated oven at 45°C for 48 h. All the dried starch films were preserved in a humidity chamber (25°C, RH = 53%) for further testing.

### Optical properties

The transparency of the films was determined by measuring their light absorption at wavelength of 600 nm using a UV-Visible spectrophotometer Shimadzu 1601 PC (Tokyo, Japan), according to the method described by Maran et al. (2013) [14]. The film specimens were cut into strips (1×4 cm) and placed directly in the spectrophotometer test cell. Air was used as reference. Opacity was expressed as absorbance units per thickness unit. All of these samples were carried out in triplicate.

### Mechanical properties

A TA. XT Plus Texture Analyzer (Lloyd Instruments, West Sussex, England) was used to determine the tensile strength and percentage of elongation at the break. Film specimens were tested as suggested by Mehyar et al. (2012) with some modifications and the tests were carried out according to the ASTM D828–97 standard test methods (ASTM, 1997) [15]. CS/CaCO_3_ nanoparticles composite films were cut into strips (1×10 cm). The clamp distance was 20 mm and the draw rate was 100 mm/min. Tensile strength (MPa) was calculated by dividing the maximum load by a cross-sectional area of the film. Percentage of elongation at the break was expressed as a percentage of change of the original length of a specimen between grips at the break. Before the testing, the strips were preconditioned at 67% RH for 48 h at room temperature (25±1°C). All samples were carried out in triplicate.

### Measurement of water vapor permeability (WVP)

Before the testing, the films were conditioned at 25°C for 48 h in a desiccator with a relative humidity of 67%. Circular film samples were placed over the mouth of the test cup and sealed by melted paraffin in the desiccator. The test cup was about 10 mm in diameter. Anhydrous calcium chloride (0% RH) was placed inside the test cup while a saturated sodium chloride solution (75% RH) was placed in the desiccator. The change in the weight of the cups was measured every 12 h over two days. The gravimetric method was used to determine WVP of CS/CaCO_3_ nanoparticles blend films as suggested by Liu et al. (2005). The WVP was calculated as follows:
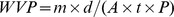
where d is film thickness (m), m is the weight increment of the cup (g), A is the area exposed (m^2^), t is the time lag for permeation (h), and P is water vapor partial pressure difference across the film (Pa). All samples were carried out in triplicate.

### Scanning electron microscopy (SEM)

The films’ external surfaces and cross sections were observed with a JSM-5610LV SEM (JEOL, Tokyo, Japan), respectively, according to the description of De la Caba et al. (2012) and Garg et al. (2007) [16]–[17]. Prior to the observation, the external surfaces were sputter-coated with a gold layer. To observe the cross section, the films were frozen in liquid nitrogen and then fractured immediately. The fracture surfaces were sputtered with gold and then photographed.

### Differential scanning calorimetry (DSC) analysis

DSC experiments were carried out using DSC1 (METTLER TOLEDO, Switzerland). The calorimeter was calibrated with indium (melting point 156.6°C, heat of fusion 28.5 J/g). The DSC runs were operated under nitrogen gas atmosphere (30 mL/min) and an empty pan was used as the reference. The film samples, approximately 3 mg, were hermetically sealed in aluminum pans. The pans were heated from 15°C to 300°C at the scanning rate of 10°C/min. The DSC thermograms were evaluated to characterize the onset, peak and end temperatures and the enthalpy changes of the phase transitions.

### X-ray diffraction

The crystalline structure of the film samples was analyzed by Philips PW1710 (Philips, Holland), provided with a tube, a copper anode, and a detector operating at 45 kV and 30 mA within 2θ from 4 to 40° with a 0.02° step size.

### Statistical analysis

The experiment data were subjected to statistical analysis using SPSS 17.0 (SPSS Inc., 160 Chicago, USA). The data were analyzed using analysis of variance (ANOVA) using the Origin Pro 7.5 statistics program and expressed as mean values ± standard deviation. Differences were considered at a significant level of 95% (p<0.05).

## Results and Discussion

### WVP and Optical properties of films

The opacity of CS/CaCO_3_ nanoparticles composite films are shown in Table 1. The film opacity values were used to assess the transparency of the films. As we can see, the pure CS films had the lowest opacity. Film opacity increased significantly (p<0.05) with an increase in CaCO_3_ nanoparticles concentration in corn starch formulations. A similar tendency was reported by Mbey et al. (2012) for cassava starch-kaolinite composite films; they reported that when talc was added to the plasticized cassava starch matrix, there was a reduction of transmittance [18]. The possible reason for higher opacity value in films is due to the fact that mean particle size of the nanoparticles is almost similar to the size of the interspaces in starch film. When the light passes through these films, a much lower extent of light is transmitted through the film, which results in higher opacity value [19]. Bodirlau et al. (2012) reported that the increase in ordered zones led to reduce absorbance and increase film transparency [20]. In general, higher light absorbance of films related to desirable properties of food packaging since it was an excellent barrier to prevent light-induced lipid oxidation.

**Table 1:** Water vapor permeability (WVP) and optical properties of corn starch (CS) based films with CaCO_3_ nanoparticles (Ca)^g^.

Films	d/mm	Opacity	WVP(10^−10^ g Pa^−1^ s^−1^ m^−1^)
CS	0.173±0.01^a^	1.19±0.11^f^	5.36±0.13^a^
CS+0.02% Ca	0.187±0.01^a^	1.29±0.21^e^	3.79±0.15^b^
CS+0.04% Ca	0.166±0.02^a^	1.45±0.16^d^	2.98±0.10^c^
CS+0.06% Ca	0.179±0.01^a^	1.62±0.15^c^	1.58±0.09^e^
CS+0.1% Ca	0.189±0.01^a^	1.94±0.06^b^	2.32±0.17^d^
CS+0.5% Ca	0.157±0.03^a^	2.23±0.16^a^	2.65±0.07^d^

g values correspond to the mean±standard deviation. Values within each column followed by different letters indicate significant differences (p<0.05).

The water vapor permeability (WVP) of corn starch/CaCO_3_ nanoparticles blend films is shown in Table 1. WVP of the films is important when it is applied as packaging materials. In such cases one of the functions of the film is to avoid, or at least to decrease, moisture transfer between the food and the surrounding atmosphere; WVP should be as low as possible. As can be seen from Table 1, the pure CS films had the highest WVP (5.36×10^−10^ g Pa^−1^ s^−1^m^−1^), which was significantly higher than starch composite films containing CaCO_3_ nanoparticles. When the content of CaCO_3_ nanoparticles was 0.06%, the blend films had the lowest value of WVP.

The enhancement in water vapor resistance with the addition of CaCO_3_ nanoparticles can be due to their nanometric size, which increases the surface volume ratio and promotes a better dispersion of the nanoparticles in the starch matrix. The well distributed nanoparticles can generate a curved path and force water molecules to flow through the composite in a tortuous path, decreasing their diffusion through the film. In addition, the CaCO_3_ nanoparticles are less hydrophilic than starch, making the film more hydrophobic. Muller et al. (2011) have also observed a decrease in the WVP of the films caused by the incorporation of nanoclay; they attributed this behavior to the tortuous paths available for water vapor diffusion [21]. It had been previously reported that the nanoparticles could prevent the formation of hydrogen bonding between starch molecules, giving rise to a more compact structure with smaller inter-chain spaces that can reduce the water vapor diffusion through the film. In general, CS/CaCO_3_ nanoparticles composites films showed better barriers to water vapor.

### Mechanical properties of the films

The effect of CaCO_3_ nanoparticles content on mechanical properties is presented in Table 2. Food packaging generally requires resistance to high stress with deformation according to the intended application. The pure CS films had the lowest mechanical properties. The addition of CaCO_3_ significantly improved tensile strength, elongation at the break and Young’s modulus, which increased from 1.40 to 2.24 MPa, 79.21 to 118.98% and 1.82 to 2.41 MPa, respectively. The tensile strength of the films increased with the increasing of the CaCO_3_ nanoparticles content up to 0.06% and then decreased as the increasing of the nanoparticle content went on. It seemed as if loading more than 0.06% of the nanoparticles did not lead to a greater effect because of the phase separation between the nanoparticle aggregates and starch matrix. The results in this study were in line with Min Wu et al. (2009) who reported that SiO_2_ nanoparticles, as a filling agent, could effectively improve the tensile properties of starch films [22]. The increased mechanical properties might be attributed to the well dispersion state of nanoparticles and the interactions between CaCO_3_ nanoparticles and chain segments of corn starch, which reduced chain mobility and hence improved macroscopic rigidity of CS/CaCO_3_ nanoparticles composite films. Chivrac (2008) reported that the nanocomposites exhibited a remarkable improvement in the mechanical properties especially in the Young’s modulus and this was due to the filler surface-polymer chain segments interactions which reduced chain mobility and hence improved macroscopic rigidity [23]. Nanofillers act efficiently as matrices reinforcement, but only if they are well dispersed, so the interface with the matrix would be maximized.

**Table 2:** Mechanical properties of corn starch (CS) based films with CaCO_3_ nanoparticles (Ca)^g^.

Films	Tensile strength/MPa	Elongation at break/%	Young’s modulus/MPa
CS	1.40±0.21^c^	79.21±7.69^f^	1.82±0.11^d^
CS+0.02% Ca	1.86±0.01^b^	107.67±2.23^d^	1.97±0.08^c^
CS+0.04% Ca	1.94±0.11^b^	113.32±5.49^b^	2.13±0.08^b^
CS+0.06% Ca	2.24±0.09^a^	118.98±3.53^a^	2.41±0.07^a^
CS+0.1% Ca	1.93±0.16^b^	108.91±4.20^c^	1.92±0.07^c^
CS+0.5% Ca	1.83±0.04^b^	105.57±2.63^e^	1.84±0.07^d^

g values correspond to the mean±standard deviation. Values within each column followed by different letters indicate significant differences (p<0.05).

### SEM image of CS/CaCO_3_ nanoparticles blend films

**Figure 2:**
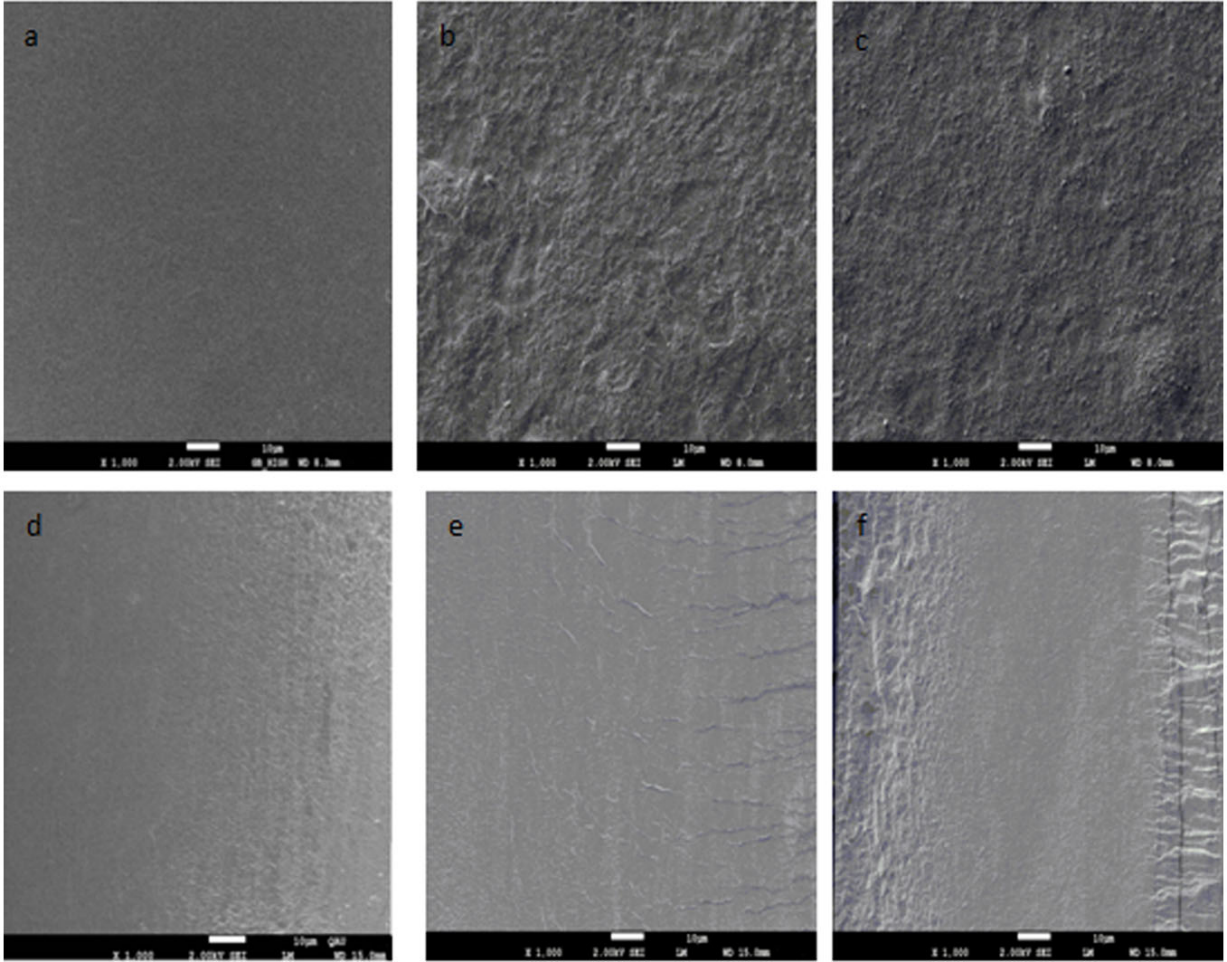
SEM micrograph from surface of corn starch (CS) films (a), composite films with (b) 0.06%, (c) 0.5% CaCO_3_ nanoparticles; and fracture of CS films (d), composite films with (e) 0.06%, (f) 0.5% CaCO_3_ nanoparticles.

The SEM image of CS/CaCO_3_ nanoparticles blend films is shown in Fig. 2. As can be seen from Fig. 2, the surface of starch film without nanoparticles was smooth, however, the films containing CaCO_3_ nanoparticles (Fig. 1b and c) showed roughness, especially on the films containing 0.5% content CaCO_3_ nanoparticles, which exhibited many protuberances or micro-scaled particles indicating the phase separation. The phase separation could weaken the interface adhesion between nanofiller and matrix leading to decreasing tensile strength of the films with higher content of the nanoparticles (Table 2). The tuber on the image suggested that the nanoparticles are uniformly scattered in the CS matrix. De Melo et al. (2011) stressed that the surfaces of starch-nanoclay films showed less smooth than that of the starch films [24]. The incorporation of nanoparticles resulted in a density network structure, which was a good indicator of high tensile strength as shown in Table 2.

The cross-section images of films prepared with different CaCO_3_ nanoparticles ratios are shown in Fig. 2. Pure starch films (Fig. 1d) showed a continuous and smooth aspect, indicating the integrity of the structure. This result was consistent with Mali et al. (2002), who reported that a compact and homogeneous matrix was observed in yam starch-based films with glycerol produced by casting [25]. The composite films with 0.06% CaCO_3_ nanoparticles (Fig. 1e) showed a rough fracture, while several micro-folds could be observed in the cross-section, which might be attributed to the small addition of nanoparticles. The films needed more energy and had larger tensile strength. Furthermore, the long and deep cracks could be found in 0.5% CaCO_3_ nanoparticles (Fig. 1f), which indicated the formation of a more fragile structure in these cases. The results may be due to molecular irregularity increasing with an increment of particle concentration.

### Thermal properties of nanoparticles/starch blends

The effects of CaCO_3_ nanoparticles on the thermal properties of nanoparticles/starch blends were evaluated by DSC. Table 3 shows the thermal parameters of various starch/nanoparticles blend films.

**Table 3:** Thermal properties of corn starch (CS) based films with CaCO_3_ nanoparticles (Ca)^g^.

Film Sample	Onset temperature(°C)	Melting temperature(°C)	Melting enthalpy (J/g)
CS	191.75±0.08^e^	217.11±0.08^e^	17.85±0.08^a^
CS+0.02% Ca	204.07±0.11^c^	219.40±0.11^d^	25.45±0.07^d^
CS+0.04% Ca	194.90±0.11^d^	225.03±0.07^c^	30.02±0.08^e^
CS+0.06% Ca	215.08±0.13^a^	236.83±0.09^a^	31.09±0.09^f^
CS+0.1% Ca	209.73±0.07^b^	228.27±0.08^b^	23.75±0.09^c^
CS+0.5% Ca	204.25±0.07^c^	219.30±0.01^d^	20.55±0.08^b^

g values correspond to the mean±standard deviation. Values within each column followed by different letters indicate significant differences (p<0.05).

From Table 3, we observed that there was a single endothermic transition between 190 to 240°C. When CaCO_3_ nanoparticles were added to the films, the transition shifted to higher values. This change may be due to the presence of smaller and more irregular CaCO_3_ nanoparticles in nanocomposites than those in the control films. Both onset and melting temperature of the nanocomposite films were higher than those of the corn starch films. The melting temperature of starch/nanoparticles blend films was highest in the presence of 0.06% CaCO_3_ nanoparticles. This could be due to the fact that the nanoparticles were well distributed in the starch matrix, which increased the contact area between the nanoparticles and the matrix and consequently increased the compact structure of the films, which required a higher temperature to disorganize. A similar reasoning was offered by Selene Aila-Suárez et al. (2013) [26], who found that cellulose nanoparticles increased the contact area between both polysaccharides, requiring a higher temperature to melt the structure. The melting enthalpy of CS film was 36.07 J/g. Likewise, Selene Aila-Suárez et al. (2013) also reported that the ΔH value of CS film was 28.25 J/g [26]. With the increase of CaCO_3_ nanoparticles, the melting enthalpy of films became higher than pure corn starch; this may be due to the interactions between CaCO_3_ nanoparticles and chain segments of corn starch, which increased the crystallinity of the film. It could be deduced that the higher the values of the melting enthalpy, the higher compatibility of CS/CaCO_3_ nanoparticles.

### Amorphous/crystalline nature

**Figure 3:**
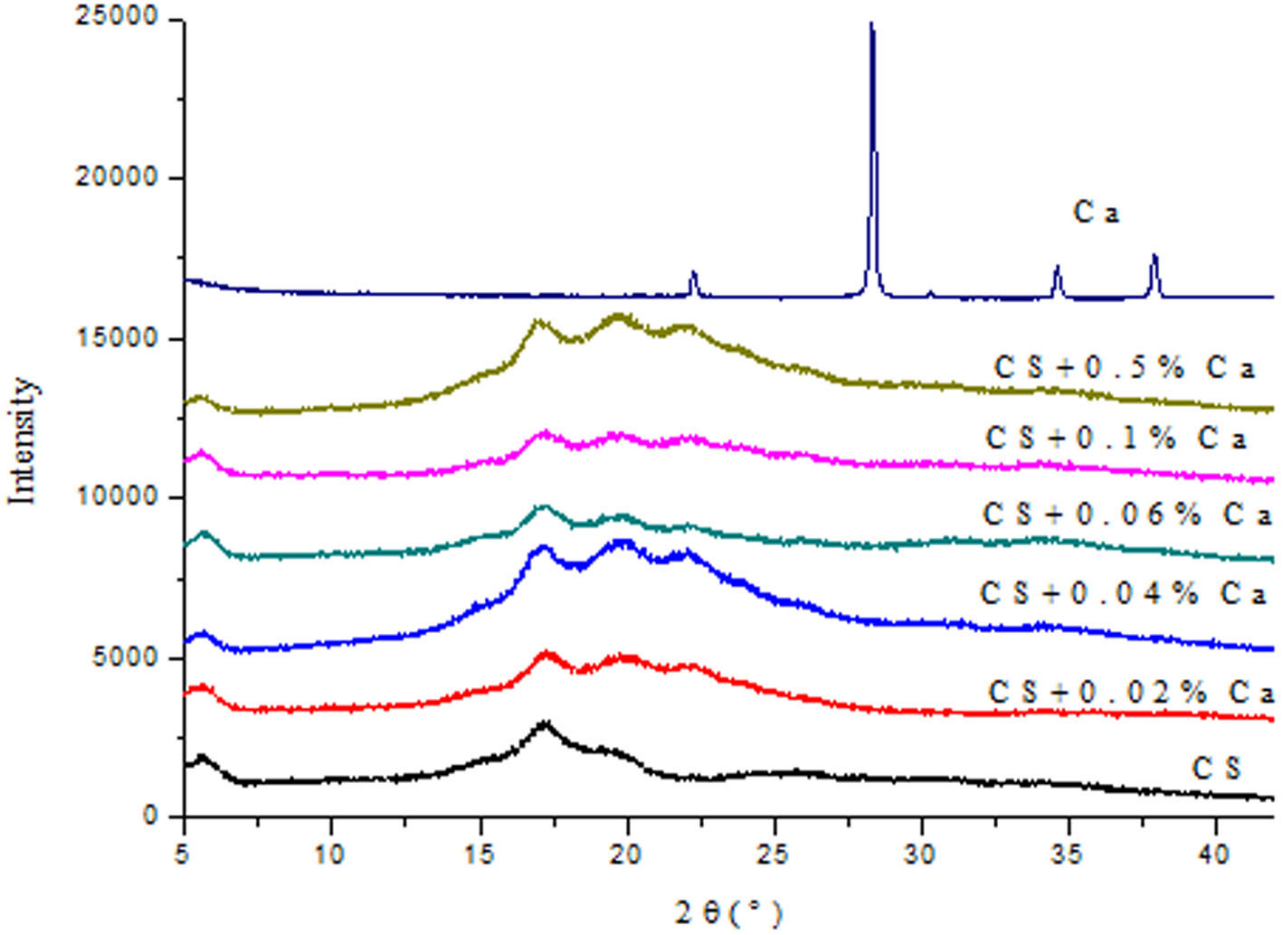
X-ray diffraction patterns spectra of CaCO_3_ nanoparticles (Ca) and corn starch (CS) films with 0, 0.02, 0.04, 0.06, 0.1, 0.5% w/w Ca.

The diffraction patterns of CaCO_3_ nanoparticles and corn starch films in the presence and absence of the nanoparticles in between 5°(2θ) and 40°(2θ) are shown in Fig. 3. As can be seen from Fig. 3, the X-ray diffraction pattern of the pure CS film presented low intensity, narrow diffraction peaks and low crystallinity. CS film spectra showed the main characteristic peaks at 5.6° and 17°. This diffraction pattern may be due to the strong interaction between hydroxyl groups of starch molecules that were substituted by hydrogen bonds formed between the plasticizer and starch during processing. However, the characteristic diffraction peaks of the nanocomposite films had slight changes compared to the CS matrix and pure CaCO_3_ nanoparticles. The characteristic peaks of CS/CaCO_3_ nanoparticles film that can be detected at 5.6°, 17°, 19.5° and 22° assigned them to a B and V type structure. The peak of CaCO_3_ nanoparticles at 17.5° disappeared in the nanocomposite films, that may be due to the good compatibility between corn starch and CaCO_3_ nanoparticles. A widening in the peaks can be observed in Fig. 3. Ungar (2004) stressed that a broadening X-ray peak indicated that crystal lattice became imperfect, according to the theory of kinematical scattering; otherwise, peak broadening is caused by small crystal size [27]. As was expected, the addition of nanoparticles modified the peak intensity of films, which became stronger than that of CS films.

## Conclusions

The mechanical properties (tensile strength, elongation at the break and Young’s modulus) increased with the addition of CaCO_3_ nanoparticles, indicating that CaCO_3_ nanoparticles could be used to greatly improve film strength and flexibility. When CaCO_3_ nanoparticles were added to CS the film at 0.06% level, the WVP of the films was significantly decreased. The result showed that the water barrier properties of the CS film were obviously improved by the incorporation with CaCO_3_ nanoparticles into CS films, suggesting that the smooth and compact structure between nanoparticles and CS was formed, which can be confirmed by the SEM image. The addition of nanoparticles increased the melting temperature of films by DSC. X-ray diffraction results showed that CaCO3 nanoparticles and corn starch matrix had good compatibility. Therefore, it can be concluded that the CaCO_3_ nanoparticles and CS would be an attractive method to develop new edible films. It was observed from this work that the content of CaCO_3_ nanoparticles at 0.06% may be the best option for the desirable properties of edible films.
